# Metagenomic and gene expression patterns in declining commercial honey bee colonies

**DOI:** 10.1038/s41598-026-42605-w

**Published:** 2026-03-03

**Authors:** Anthony Nearman, Zachary S. Lamas, Elina L. Niño, Julia Fine, Christopher Mayack, Arathi Seshadri, Dawn Boncristiani, Wei-Fone Huang, Jay D. Evans, Yan Ping Chen

**Affiliations:** 1 USDA-ARS Bee Research Lab, BARC-East Bldg. 306, Beltsville, MD 20705 USA; 2 Department of Biology, University of Maryland, Baltimore County, Baltimore, MD USA; 3 Department of Entomology and Nematology, University of California Davis, One Shields Avenue, Davis, CA 95616 USA; 4 Invasive Species and Pollinator Health Research Unit, USDA-ARS, 3026 Bee Biology Rd, Davis, CA 95616 USA; 5 Department of Biology, William Paterson University, Wayne, NJ 07470 USA; 6 USDA-ARS, Pollinator Health in Southern Crops Ecosystems Research Unit, Stoneville, MS 38776 USA; 7 School of Agriculture and Natural Resources, Kentucky State University, 400 E. Main St, Frankfort, KY 40601 USA

**Keywords:** Agriculture, Gene expression, Metagenomics, Pollination, RNA-sequencing, Virus, Ecology, Ecology, Microbiology, Molecular biology

## Abstract

Managed honey bee colonies (*Apis mellifera*) in the US continue to experience high overwinter loss rates driven by parasites, pathogens, poor nutrition, and pesticides. To mitigate these losses, inspection and monitoring are critical for identifying traits of colonies in decline and potential causal factors. In this study, we apply molecular methods to associate potential causative agents with colonies in various stages of decline. Initially, we investigated in-hive bee metagenomic RNA isolated from 15 colonies across seven managed operations in California whose adult bee and brood populations were classified as Strong, Medium, or Weak in strength. We discovered that Weak colonies harbored 2.2- and 3.6- fold more viral species than Medium and Strong colonies, respectively, as well as larger viral read pools despite similar library sizes. They also displayed higher nucleotide variation in *Varroa*-vectored viruses, indicating associations with high mite populations. When investigating differences in host gene expression, we discovered an upregulation of immune-related pathways in Weak colonies relative to Strong. Specifically, Weak colonies upregulated genes related to wound healing, phagocytosis, oxidative stress resistance, apoptosis, and RNA interference. Most antimicrobial peptides were upregulated in Weak colonies, although *defensin1* was significantly higher in Strong colonies, along with several detoxification enzymes and the royal jelly peptide *apisimin*. Weak colonies also showed an upregulation of transcripts tied to abnormal protein digestion. The low levels of viral replication and fewer species of mite-vectored viruses in Strong colonies may be due to successful *Varroa* management. Strong colonies also displayed upregulated levels of nine different ubiquinone transcripts, arguably reflecting increasing longevity or a younger in-hive population compared to Weak colonies. Overall, these results provide a detailed account of viral metagenomics and associated host responses, providing new insights into the mechanisms underlying honey bee colony decline under comparable management conditions.

**Supplementary Information:**

The online version contains supplementary material available at 10.1038/s41598-026-42605-w.

## Introduction

Managed honey bees in the US continue to experience high rates of colony loss^1–3^, contributing to economic concern among stakeholders^4,5^. Researchers are focused on investigating the underlying drivers of loss and developing approaches to monitor colony health in real time. While existing health management measures have contributed to reducing losses^6^, additional diagnostic tools are needed to further improve these results. Further, continued monitoring remains essential to detect emerging pathogens and more virulent strains of existing pathogens^7^.

Metagenomic analysis represents a critical first step in developing new diagnostic tools^8–10^. This approach facilitates the discovery and characterization of novel pathogens^11,12^, reveals the combinations of viruses and parasites present^8,9^, and maps sequence divergence among viral quasi-species^13^. With improved sequencing technologies^14^, genomic^15^ and hologenomic (host plus metagenome)^16–18^ resources, researchers are better positioned to reveal causal factors, a key step toward understanding and potentially mitigating colony losses. Another promising approach to improving colony health diagnostics involves analyzing the host’s RNA (transcriptome)^19,20^. As viral titers alone are not always predictive of colony loss^21,22^, drawing associations between combinations of specific viruses and changes to a host’s transcriptome may reveal gene targets related to tolerance, resistance, or susceptibility to infection. Incorporating such targets into existing rapid diagnostics of viruses could increase predictive power of colony health assessment. This could include, but not limited to, genes specific to immune function.

In this study, we assess viral and transcriptomic differences across honey bee colonies classified in the field as Weak, Medium, or Strong based on population strength. To do so, 15 colony-level samples from seven different commercially managed beekeeping operations were collected and RNA sequencing was performed. The resulting libraries were aligned to host and known cellular parasite genomes and quantified. Remaining sequenced reads were assembled into contigs and identified. Accession sequences of identified viral contigs were downloaded, and libraries were aligned to measure diversity and quantify read counts. The results indicate combinations of viruses and host transcript expression that are associated with colony strength. This study details key biological differences among colonies in varying stages of decline.

## Results

**Fig. 1:**
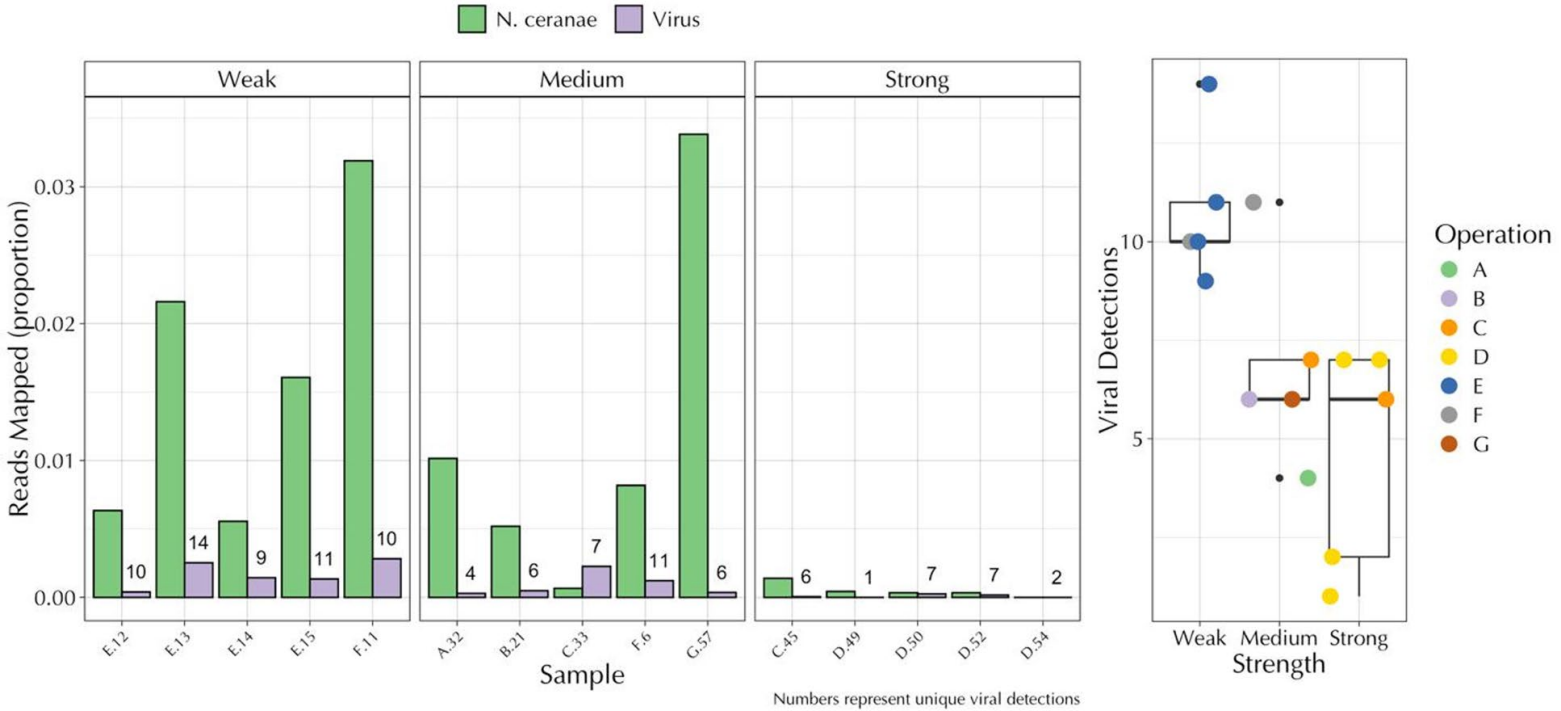
Proportion of sequenced reads mapped to viruses and Nosema ceranae by colony strength. Numbers and box plot represent unique viral detections. Viral detections with less than 25% genome coverage were removed when counting the number of viruses. Colonies are organized by their adult bee and brood frame population strength (Weak, Medium, Strong) and source beekeeping operation (A: G).

*Colonies observed to have Strong populations presented fewer viruses*,* viral transcripts*,* and Nosema transcripts compared to Weak and Medium Strength colonies.* A declining trend was observed for the within-library proportion of mapped virus reads for Weak (1.7e-03 ± 4.3e-04), Medium (9.3e-04 ± 3.7e-04), and Strong colonies (9.7e-05 ± 5.0e-05) (Fig. 1). Accounting for the number of unique viruses with > 25% genome coverage revealed the same trend, with Weak colonies containing an average of 10.8 ± 0.9 unique viral alignments, Medium colonies 6.8 ± 1.2, and Strong colonies 4.6 ± 1.3 (Fig. 1). Among the 22 unique viral accessions detected across all colonies, the odds of detection among Weak colonies were 3.6 times (95%CI = 1.8–7.4, z-value = 4.3, *p* < 0.001) higher compared to Strong colonies on average, and 2.2 times more (95%CI = 1.1–4.2, z-value = 2.7, *p* < 0.02) compared to Medium colonies. No significant difference was observed between Medium and Strong colonies in the number of viruses. Transcripts mapping to the *Nosema (Vairimorpha) ceranae* genome displayed a similar declining pattern across colony strengths (Fig. 1). The mean number of reads per library did not differ substantially among colony strength groups, with values falling within one standard error of each other (Fig. S1).

**Fig. 2:**
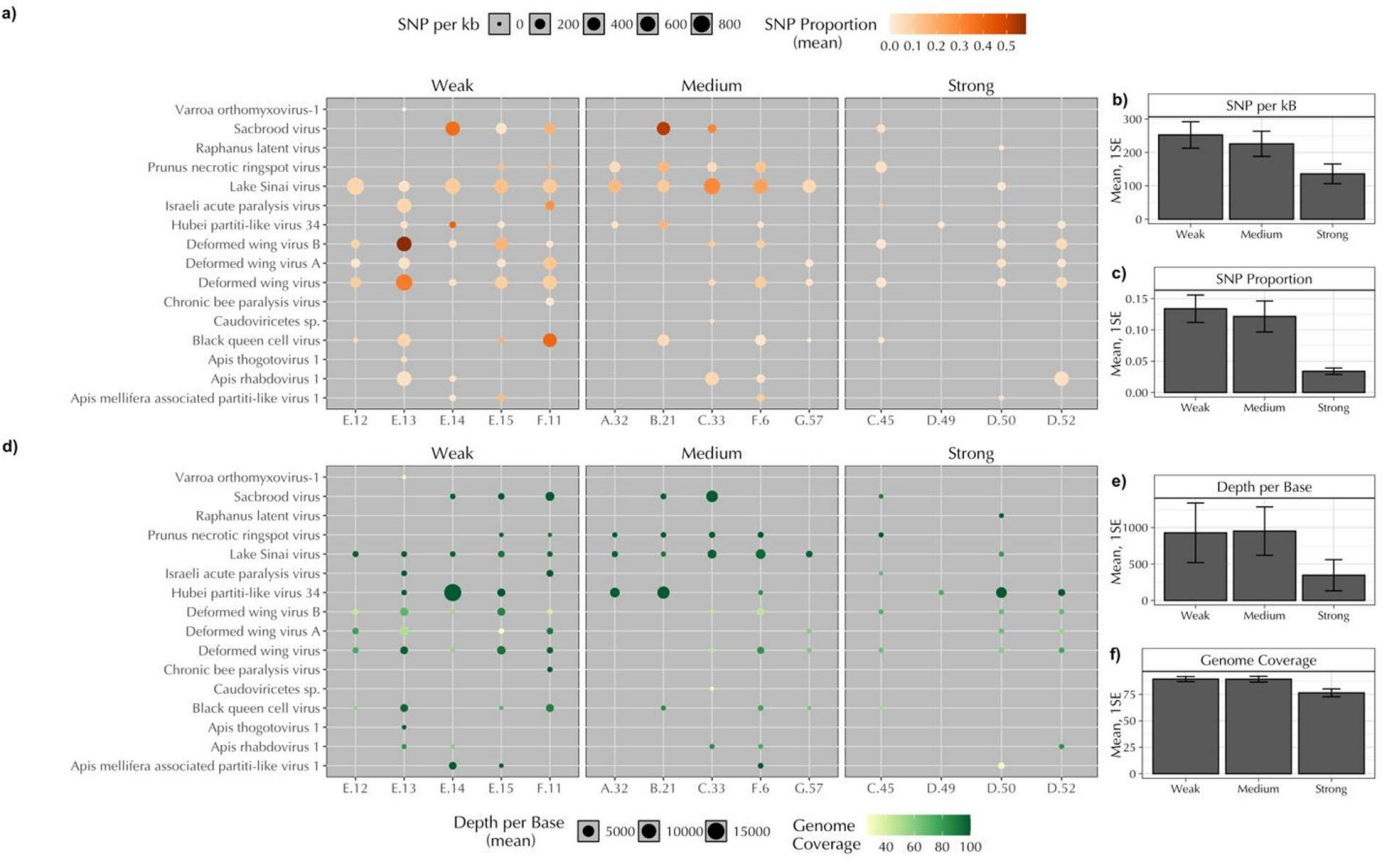
Measures of viral presence in a host. Single Nucleotide Polymorphism (SNP) count per kilobase of viral genome and their proportional representation in the aligned read pool (**a**); Mean and 1SE of overall SNPs per kilobase (**b**); Mean and 1SE proportional SNP representation in the read pool (**c**); Mean alignment depth per base and proportion of genome coverage for aligned viruses (**d**), Mean and 1SE read depth per virus per library (**e**); and Mean and 1SE genome coverage per virus per library (**f**). Detections with less than 25% genome coverage for all libraries were removed. *Apis mellifera* filamentous virus (AmFV) was excluded from SNP analysis due to extreme relative counts. Colonies are organized by their relative population strength (Weak, Medium, Strong) and source beekeeping operation (A: G). Numbers represent unique colony IDs.

*On a per-virus per-library basis*,* sequencing results indicate trends with colony population strength*. Among the number of polymorphic sites (variant calls with > 1% read proportion), Weak colonies displayed an average of 252.4 ± 39.6, Medium colonies 225.7 ± 37.7, and Strong colonies 135.8 ± 29.6 per viral kilobase per library (Fig. 2a-b). The proportion of reads containing polymorphisms was lower in Strong colonies (Weak = 0.134 ± 0.022, Medium = 0.121 ± 0.025, Strong = 0.034 ± 0.005, Fig. 2a and c). Mean sequencing depth per base (Weak = 927.9 ± 409.5, Medium = 952.1 ± 332.0, Strong = 345.8 ± 213.5) and the mean proportional length of coverage (Weak = 0.894 ± 0.024, Medium = 0.893 ± 0.027, Strong = 0.764 ± 0.037) also followed similar trends (Fig. 2d-f). Specific viruses over-represented in weak colonies include two variants of Deformed wing virus, Israeli acute paralysis virus, and *Apis mellifera* filamentous virus (Fig. 2, Fig. S2). A standard panel of viruses, including multiple Lake Sinai variants, indicate differences in viral titers for Black Queen Cell virus and three Lake Sinai variants, with most viruses present in all strength categories except Israeli acute paralysis virus and Kashmier bee virus (Fig. S3, Table S1).

*Clustering colonies and their associated viruses by β-actin normalized read counts indicates relationships to colony strength*. Hierarchical clustering reveals three distinct clades of colonies and two distinct clades of viruses. The first and third clades of colonies, consisting of all Weak, Medium, and 1 Strong colony, are similar in terms of co-infection levels that span both clades of viruses. One clade with striking differences between Strong colonies and others contains three variants of Deformed wing virus as well as *Apis mellifera* filamentous virus. The second clade of colonies, consisting of all Strong colonies, showed few viruses overall (Fig. S2).

*Lake Sinai viruses (LSV) assembled contigs reveal associations to multiple strains*,* particularly among Medium strength colonies*. To better understand the variation and existing evidence regarding LSV, reads that aligned to any known variant were filtered from their libraries and assembled into contigs. RNA-dependent RNA polymerase genes were then identified and extracted from contigs > 5 kb and aligned to closely related NCBI Genbank accessions. The resulting phylogenetic tree reveals three major clades between assembled contigs and previously published sequences. Six of the eleven assembled RdRp genes are nearly identical and closely related to LSV-4, while the remaining contigs indicate close relationships to LSV, LSV-1, LSV-2, LSV-3, LSV-NE, and LSV-TO. Four of the contigs that diverge from the LSV-4 clade are additional contigs from those same colonies, indicating a multi-strain infection or rapid mutation within host colonies (Fig. S4).

*Differential gene expression results suggest relationships to colony population strength*. Using an adjusted p-value threshold of < 0.01, comparisons between Weak and Strong colonies identified 776 differentially expressed transcripts, including 386 upregulated and 390 downregulated. Hierarchical clustering of colonies by their gene expression profiles indicates distinct separation by colony population strength (Fig. S5). Further, principal component analysis of the top 100 differentially expressed transcripts also reveals this separation, with 40% of the variation explained along the primary component axis (Fig. S6).

**Fig. 3:**
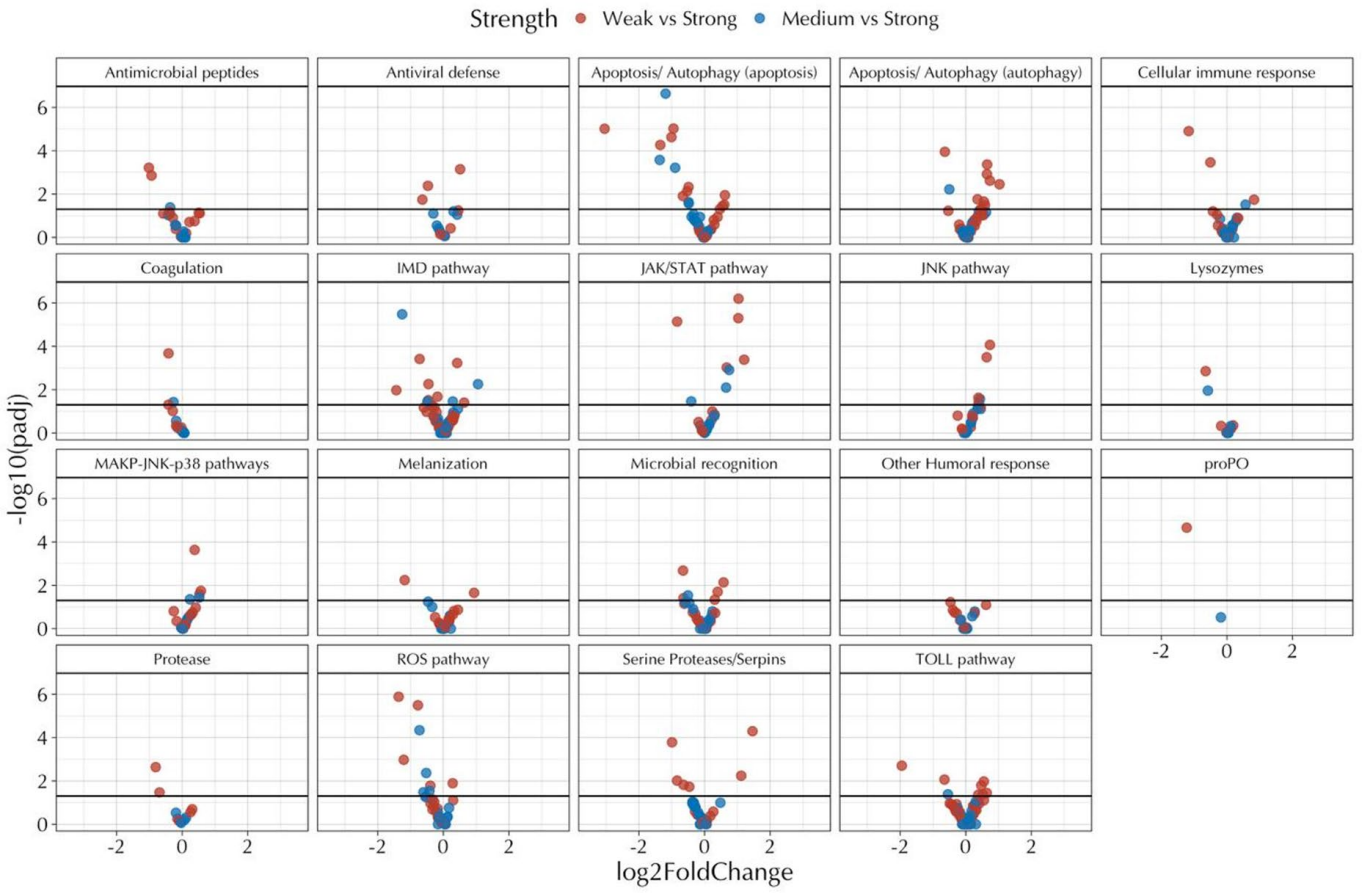
Volcano plot of differentially expressed genes curated into immune pathways. The black line represents a p-value of -log_10_(0.05). Results are displayed for pathways where at least one differentially expressed gene was present.

*Immune*,* GO*,* and KEGG pathway analyses support disease-related trends related to colony strength*. Isolating genes by their associated immune gene pathways reveal multiple targets for identifying colonies in various stages of decline (Fig. 3, Fig. S7). Several additional genes of interest displayed patterns related to colony strength. This includes nine transcripts for Coenzyme Q10 (ubiquinone) components (Fig. S8), three heat shock proteins (Fig. S9), six Cytochrome P450 genes (Fig. S10), and one major royal jelly protein (Fig. S11). Further, KEGG gene set enrichment and over-representation analysis suggest upregulated pathways within Weak colonies relative to Strong associated with increases in immune responses such as wound healing, phagocytosis, oxidative stress resistance, and apoptosis (Fig. S12-S13). These results are further confirmed through Gene Ontology gene set enrichment (Fig. S14-S16).

## Discussion

Since the outbreak of Colony Collapse Disorder (CCD) in 2006, metagenomic approaches have been used to associate causative agents with colony loss^8,22–26^. These studies tend to show an increase in viral diversity for colonies in poor health^8,9,22,27–29^. Here, we show that colonies identified as being Weak^30^ show a greater number of unique viral species and higher viral nucleotide diversity within those species (Fig. 1). Specifically, Weak colonies were more likely to contain viruses compared to Medium and Strong colonies, while Weak and Medium colonies showed higher nucleotide diversity and sequencing depth than Strong colonies (Figs. 1 and 2).

In this study, we demonstrate relationships between honey bee colony population strength and viral communities using a metagenomic approach. Weak and Medium strength colonies had more diverse viral communities compared to Strong colonies when their RNA was aligned to *de novo* assembled honey bee viruses (Fig. 1, Fig. S2). Similar relationships were observed when quantifying the overall number of polymorphic sites, sequencing depth, and genome coverage among identified viruses (Fig. 2). The prominent virus in all Medium strength colonies was Lake Sinai virus (LSV). Phylogenetic analysis of RdRp genes from LSV assembled contigs and closely related GenBank sequences indicates infection with multiple LSV strains or rapid within-host divergence, with most contigs being derived from Medium Strength colonies (Fig. S3). Last, a host differential gene expression analysis revealed relationships to colony strength that contained differences across multiple curated pathways for response to disease and detoxification (Fig. 3, Fig. S7-S16).

Viruses vectored by *V. destructor* (i.e., DWV-A, DWV-B, and IAPV) were detected at higher prevalence and reproduced at higher levels in Weak colonies compared to Medium or Strong colonies (Fig. 2, Fig. S2). This pattern likely reflects heavier mite infestation in Weak colonies. Mite stress and viral infection weaken immune responses in honey bees^31,32^, which may aid in secondary infections through non-vectored transmission routes^33–35^. In addition, prior studies on colony strength demonstrate that bees from Strong colonies better resist DWV infection and replication compared to Weak colonies^36^. The link between mite stress, vectored viruses and secondary infection is further supported by our finding that Weak colonies showed 2.2 and 3.6 times the number of viral species on average compared to Medium and Strong colonies, respectively. Weak colonies also showed higher levels of *Apis mellifera* filamentous virus and greater proportions of transcripts mapped to *N. ceranae* (Figs. 1 and 2, Fig. S2).

Our analysis of differentially expressed transcripts separates colonies based on colony strength. Significant pathways and many individual genes in Weak colonies agree with controlled studies that describe upregulated immune responses when faced with viral infection^37–41^. Genes in the antimicrobial peptide family, on the other hand, were upregulated in Strong colonies (Fig. 3). Both *apisimin* and *defensin1* are upregulated in the presence of certain neonicotinoids^42^, which coincides with the upregulation of detoxification enzymes^43^(Fig. S9). *Apisimin* was previously detected in royal jelly^44^, and at least one major royal jelly protein (*mjrp5*) was upregulated in Strong colonies (Fig. S11). Also upregulated in Strong colonies are the family of ubiquinone enzymes (Fig. S8). Artificial treatment with these proteins has been demonstrated to increase longevity for bees in controlled studies^45^. Further, ubiquinone transcript abundance decreases with chronological age and task performance^46^, suggesting these results reflect either a younger cohort of in-hive bees or a lack of precocious foragers in Strong colonies. Precocious foragers have been observed in bees infected with *N. ceranae*^47^ and Deformed wing virus^48^, which we observed at higher levels and greater prevalence among Weak and Medium strength colonies.

Differential transcript abundance of specific immune pathway members with respect to colony strength may be used to predict colonies at risk of decline without the need for extensive differential gene expression analysis. Alongside qPCR quantification of a standard panel of viruses, these gene targets may improve the accuracy of future colony health diagnostic efforts and indicate where preventative measures can be used to save colonies.

Our analysis reveals an association between field measures of colony strength, viruses, and host gene expression. Differences in viral populations can be seen across all levels of colony strength. Weak and Medium strength colonies also displayed upregulated immune pathways and genes relative to Strong colonies. Strong colonies, however, had fewer viruses and lower viral replication overall, coupled with upregulation of several immune-related genes as well as genes for detoxification enzymes. Despite earlier suggestions, these upregulated responses may be the signs of a successful immune response and/or exposure to certain acaricides^49^, which agrees with the assertion of variation in successful mite control among all colonies. This work provides detailed insights into the viral dynamics that can occur within honey bee populations. It also indicates the need for improved accuracy of health diagnostic techniques beyond a standard panel of viruses, as the number of detections did not differentiate between all levels of decline. Monitoring virus levels and transcript abundances for certain indicators of host health can be a useful tool for enhancing diagnostic accuracy and serve as valuable indicators of host health, supporting efforts to prevent disease and colony loss.

## Methods

### Honey bees

In February 2023, a field investigation was conducted in California in response to reports from commercial beekeepers regarding significant colony losses. Colonies were field inspected by researchers and categorized as Strong, Medium, or Weak based on two main criteria: the size of the adult bee population and the number of brood frames within each colony^30^. A brood frame was considered “full” if more than half of it was covered with brood. Additionally, the presence of the queen was confirmed for each colony to ensure that the colony’s scores did not reflect queen failure (Supplemental Table 1).

For each colony, a brood frame covered with adult bees was carefully removed during sampling. Adult bees were gently shaken into an alcohol-cleaned plastic pan to minimize contamination and guided into one corner of the pan to facilitate collection. Bees were then collected using clean 50 mL Falcon tubes, filling each tube completely. Two tubes were collected per colony, with approximately 100–120 worker bees per tube. Collected samples were immediately placed into a container with dry ice. Once all the samples were collected, they were shipped back to the laboratory under dry ice conditions to ensure sample integrity during transit. Upon arrival at the lab, the samples were immediately transferred to a −80 °C freezer for later analysis. A graphical abstract depicting the overall experimental design is included in the supplemental figures (Fig. S17).

### Virus and mRNA enrichment from collected worker bees

Frozen worker bees were homogenized in liquid nitrogen with ceramic mortars and pestles to yield a fine powder. A total of 0.5 g of ground tissue was transferred into a 2.0 mL microcentrifuge tube containing 750 µL of 0.2× PBS and homogenized using a FastPrep system (Fisher Science) for 40 s. For each sample, four tubes (totaling 2.0 g) were prepared, and the homogenates were pooled into a 15 mL centrifuge tube.

The pooled bee homogenate was centrifuged at 3,000 × g for 10 min, and the supernatant was filtered through a 0.8 μm syringe filter to remove debris and large particles. The filtered bee homogenate was concentrated using a 30 kDa ultrafiltration tube (Amicon, Millipore) by centrifugation at 20,000 × g for 20 min. The filtrate (< 30 kDa fraction) was discarded, and additional filtered bee homogenate was added to the ultrafiltration tube. This concentration step was repeated until the retentate reached approximately 20-fold concentration.

The ultrafiltration tube was then inverted into a clean centrifuge tube, and the concentrated bee homogenate was recovered by centrifugation at 6,500 × g for 5 min. RNA was extracted from the concentrated bee homogenate using the QIAamp Viral RNA Mini Kit (Qiagen) according to the manufacturer’s protocol.

### Library preparation and sequencing

The integrity and quantity of the RNA were assessed with the RNA Nano 6000 Assay Kit on the Bioanalyzer 2100 system (Agilent Technologies). RNA samples were submitted to the University of Maryland Institute for Genome Sciences for whole-metagenome shotgun sequencing. Following DNase I digestion (Qiagen RNase-Free DNase, 1 µL of DNase I to 9 µL of RNA, incubated at 37 °C for 30 min, followed by enzyme inactivation) and ribosomal RNA depletion (Illumina Ribo-Zero Plus Microbiome rRNA Depletion Kit), cDNA was synthesized using random hexamer primers and reverse transcriptase (SuperScript™ II Reverse Transcriptase, Thermo Fisher Scientific). Illumina RNA-seq libraries were prepared with the TruSeq RNA Sample Prep Kit (Illumina, San Diego, CA) without the poly-A isolation steps. Adapters were ligated to the double-stranded cDNA, which was then purified, and library size selection was performed using AMPure XT beads (Beckman Coulter Genomics, Danvers, MA). Paired-end (100 bp) sequencing of each RNA library was conducted on the Illumina HiSeq 2500 platform.

### Metagenomic analysis

Short read libraries were analyzed using *FastQC* v0.12.1^50^ and low-quality reads and bases were removed with *trimmomatic* v0.39^51^. Reads from each library were aligned to the Amel HAv3.1 (GCA_003254395.2), *N. cerenae* (GCA_000988165.1), and *L. passim* (GCA_037349495.1) genomes, with aligned reads being quantified and removed at each step to generate a set of host and parasite cleaned libraries using *STAR* short read aligner v2.7.11b^52^. Remaining reads were normalized to a target depth of 100 using *bbnorm* from BBMap suite v39.17^53^. Normalized reads were then assembled using *SPAdes* v4.0 with the --isolate parameter^54^. The resulting contigs were filtered to a length > 500 bases and identified with the NCBI non-redundant nucleotide database using *blastn* 2.16.0 and an e-value of 1e-5^55^. BLAST results were filtered to non-viral contig IDs, which were used to generate a concatenated fasta file of non-viral contigs. This fasta of non-viral contigs was then used to remove aligned reads from the host/parasite cleaned libraries using *STAR* aligner. To remove any PCR duplicates, the remaining reads were then deduplicated using *clumpify* from the BBMap suite. These presumed virus-only, deduplicated reads were then assembled into contigs using *SPAdes* with the --rnaviral and --only-assembler parameters, with the contigs then filtered to sequences with a length > 500 bases. Contigs from the resulting filtered set were identified using *blastn* with an e-value of 1e-5 and validated using *CheckV* v1.0.3^56^. BLAST results were filtered to the top 10 viruses for each contig, and associated accession numbers were used to retrieve a non-redundant FASTA file of viral sequences with Entrez batch download^57^. This virome was then indexed and host/parasite clean libraries were aligned using *STAR* aligner. Statistics per virus were then calculated using *samtools coverage* and *mpileup* v1.22^58^ on the resulting alignment files. All downstream analyses were performed in R v4.5.1 “Great Square Root”^59^, and plots were generated with *ggplot2* v3.5.2^60^ and *ggpubr* v0.6.0^61^.

Statistical significance between virus number and colony strength was calculated using the *glm* function in base R with colony strength as the independent variable and the number of detected and undetected viruses as the dependent variable in a binomial model. Multiple comparisons were performed using the *multcomp* package^62^. Actin normalized read counts per virus were clustered and plotted using the *pheatmap* R package^63^. Actin was chosen as the housekeeping gene used to normalize read counts across libraries.

LSV-aligned reads were assembled into contigs, and their RdRP genes were extracted for multisequence alignment with *RaXML-NG* v1.2.2 under the GTRGAMMA model over 100 bootstrap replicates^64^. The resulting best tree was plotted in R with the *ggtree* and *treeio* packages^65,66^.

### Differential gene expression

Gene counts for host differential gene expression were calculated using *Salmon* v1.10.1^67^ and the Amel HAv3.1 transcriptome. Differential expression was calculated using the *DESeq2* R package v1.48.1^68^. Calculations were performed at both the transcript and gene levels. Gene set enrichment was performed using the *clusterProfiler* R package v4.16.0^69^, with access to Kyoto Encyclopedia of Genes and Genomes (KEGG) databases^70–72^.

## Supplementary Information

Below is the link to the electronic supplementary material.

Supplementary Material 1

Supplementary Material 2

Supplementary Material 3

Supplementary Material 4

Supplementary Material 5

Supplementary Material 6

## Data Availability

The RNA libraries for this study are available on NCBI under project accession PRJNA1364028 (https://www.ncbi.nlm.nih.gov/bioproject/PRJNA1364028).
