# Supplementary material for: Metagenomic and gene expression patterns in declining commercial honey bee colonies
Source: Sci Rep. 2026 Mar 3;16:11642. doi: 10.1038/s41598-026-42605-w (PMC13061895; doi:10.1038/s41598-026-42605-w)
Supplement: Supplementary file 3 — Supplementary Material 3 [file 41598_2026_42605_MOESM3_ESM.pdf]

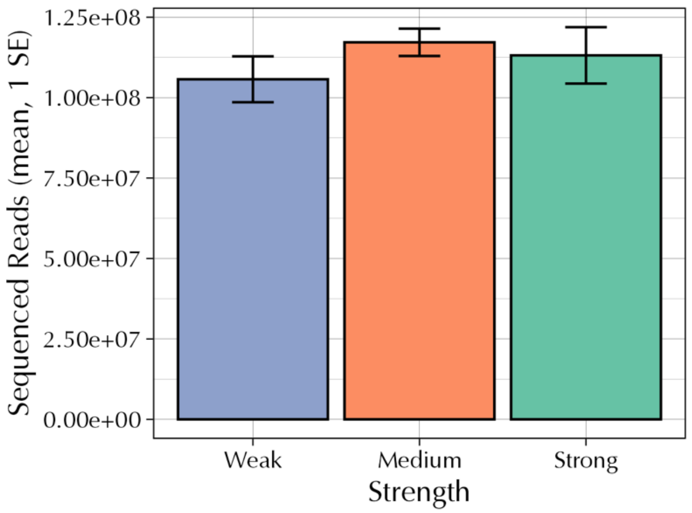

Figure S1 - Mean and 1SE of sequenced reads by colony strength. Each bar represents 5 RNA libraries of colony level brood-nest bee samples from commercial operations.

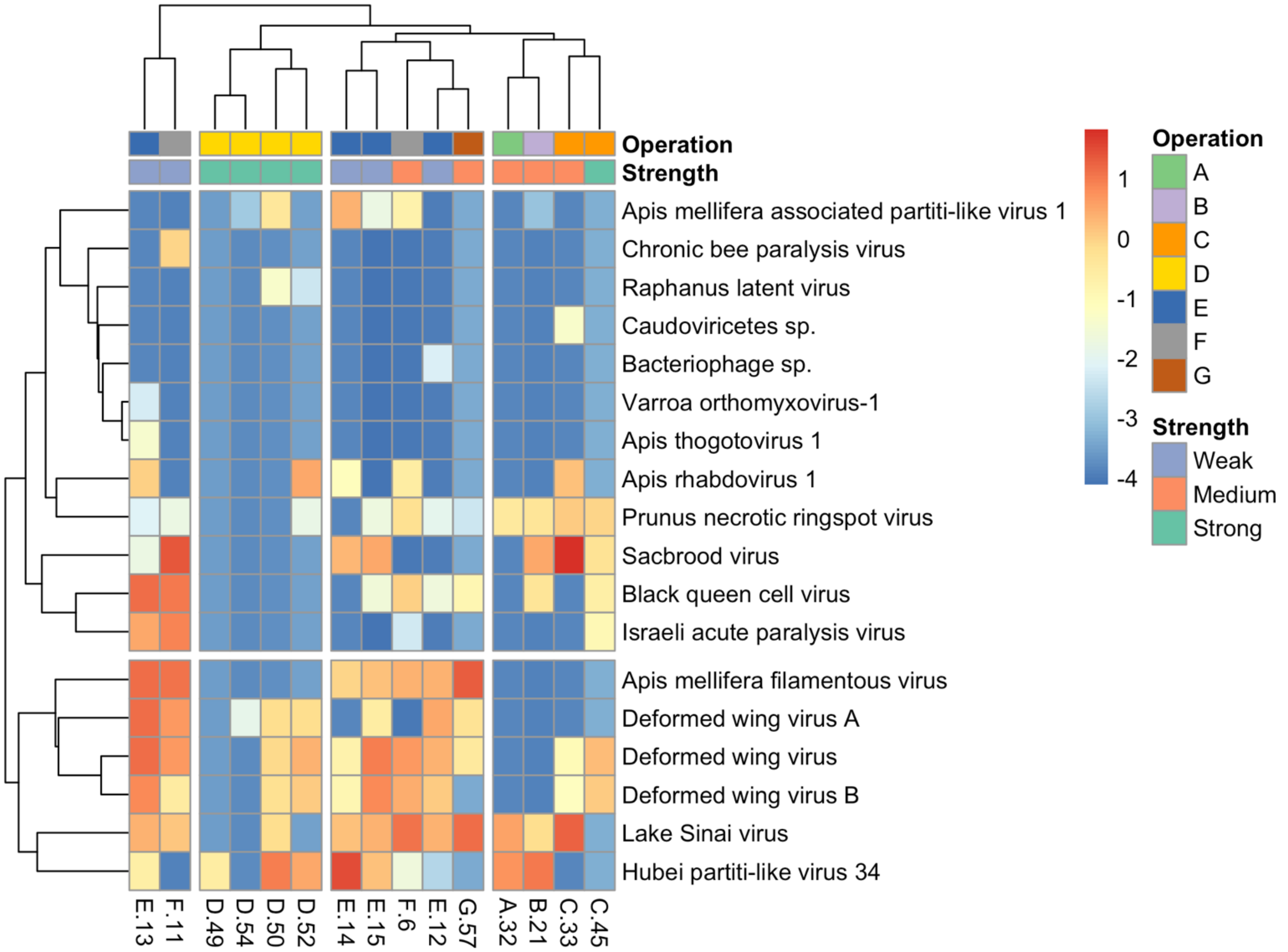

Figure S2 - Heatmap and dendrogram for read counts mapped to viral genomes normalized to within sample β-actin read counts per library (log10(viral read counts mapped to viral genomes normalized to within sample β-Actin read counts per library (log10(viral read count) – log10(β-actin read count)). Detections with less than 25% genome coverage for all libraries were removed. Colonies are organized by their relative population strength (Weak, Medium, Strong) and source

beekeeping operation (A:G). Numbers represent unique colony IDs. Clustering was performed using complete Euclidean distance.

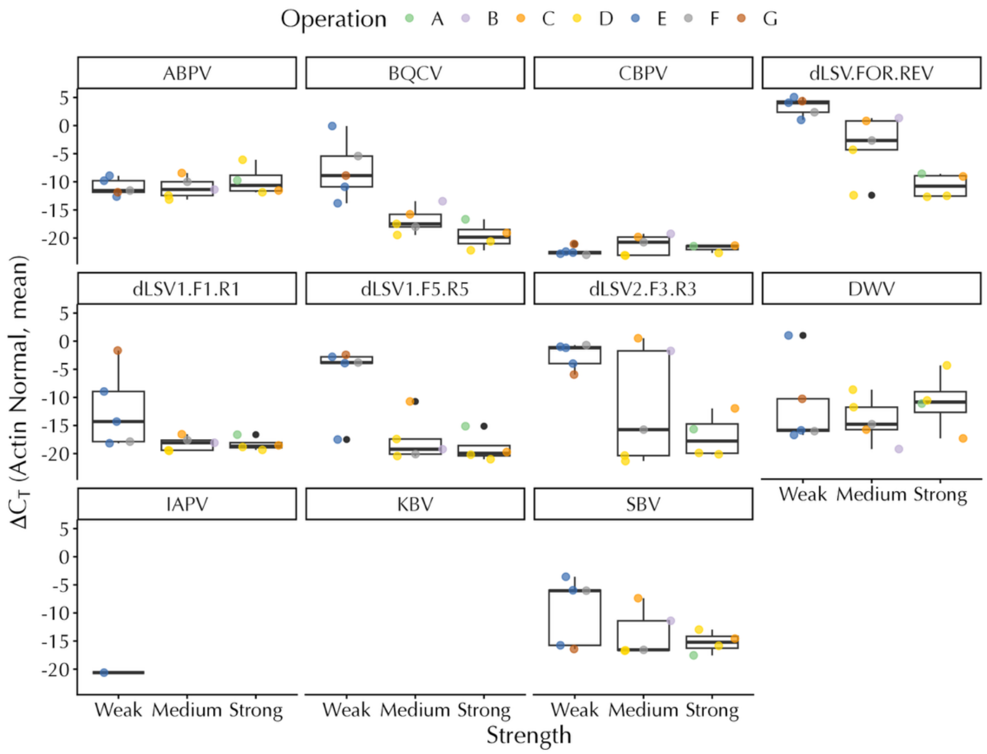

Figure S3 - qPCR results for a standard panel of viruses with the addition of four Lake Sinai variants by colony strength. Ct values are normalized by sample β-Actin measures. Values presented are the mean of three replicates for Acute Bee Paralysis virus (ABPV), Black Queen Cell virus (BQCV), Chronic Bee Paralysis virus (CBPV), Lake Sinai virus (dLSV.FOR.REV, dLSV1.F1.R1, dLSV1.F5.R5, dLSV2.F3R3), Deformed Wing virus (DWV), Israeli Acute Paralysis virus (IAPV), Kashmier Bee virus (KBV), and Sac brood Virus (SBV). Colonies are organized by their relative population strength (Weak, Medium, Strong) and source beekeeping operation (A:G).

Virus	Contrast	p adjusted
ABPV	Medium-Weak	0.994
	Strong-Weak	0.684
	Strong-Medium	0.627
BQCV	Medium-Weak	**0.007**
	Strong-Weak	**0.002**
	Strong-Medium	0.515
CBPV	Medium-Weak	0.359
	Strong-Weak	0.827
	Strong-Medium	0.795
DWV	Medium-Weak	0.787
	Strong-Weak	0.980
	Strong-Medium	0.700
SBV	Medium-Weak	0.350
	Strong-Weak	0.198
	Strong-Medium	0.879
dLSV.FOR.REV	Medium-Weak	**0.033**
	Strong-Weak	**0.000**
	Strong-Medium	**0.032**
dLSV1.F1.R1	Medium-Weak	0.112
	Strong-Weak	0.130
	Strong-Medium	0.999
dLSV1.F5.R5	Medium-Weak	**0.007**
	Strong-Weak	**0.005**
	Strong-Medium	0.896
dLSV2.F3.R3	Medium-Weak	0.124
	Strong-Weak	**0.022**
	Strong-Medium	0.508

Table S1 – Pairwise comparisons of the qPCR results for Acute Bee Paralysis virus (ABPV), Black Queen Cell virus (BQCV), Chronic Bee Paralysis virus (CBPV), Lake Sinai virus (dLSV.FOR.REV, dLSV1.F1.R1, dLSV1.F5.R5, dLSV2.F3R3), Deformed Wing virus (DWV), Israeli Acute Paralysis virus (IAPV), Kashmier Bee virus (KBV), and Sac brood Virus (SBV). Adjusted p-values represent the ANOVA and subsequent pairwise Tukey HSD tests across three replicates for each colony. Adjusted p-values in bold indicate a p<0.05.

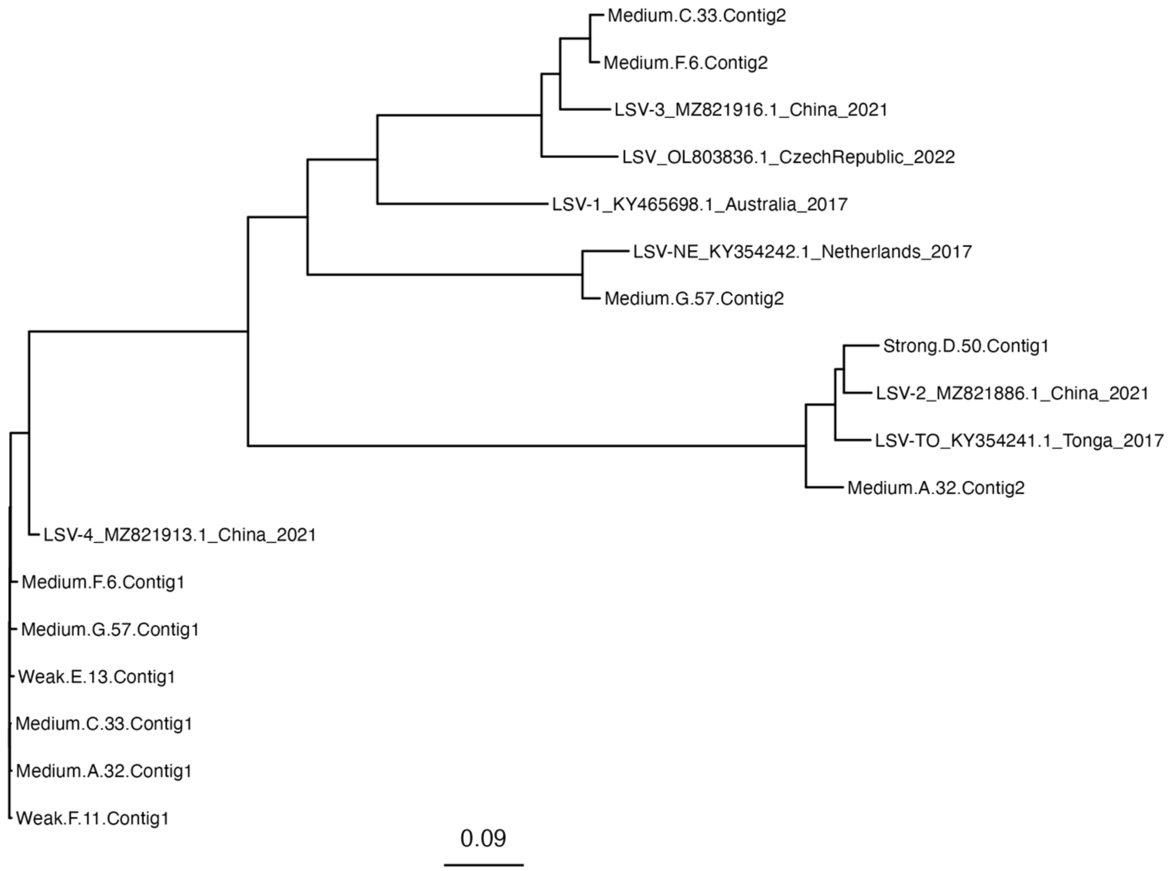

Figure S4 - Phylogeny of RNA-dependent RNA polymerase (RdRp) nucleotide sequences for LSV assembled contigs from this study (2023) and closely related NCBI Genbank sequences. Maximum likelihood trees were generated over 100 bootstrap replicates using the GTRGAMMA model. NCBI accessions were selected based on their similarity to assembled contig RdRp using blastn. Contigs are labeled by their host colony's relative population strength (Weak, Medium, Strong), source beekeeping operation (A:G), colony ID, and contig ID. Assembled LSV contigs from this study are included as supplemental material.

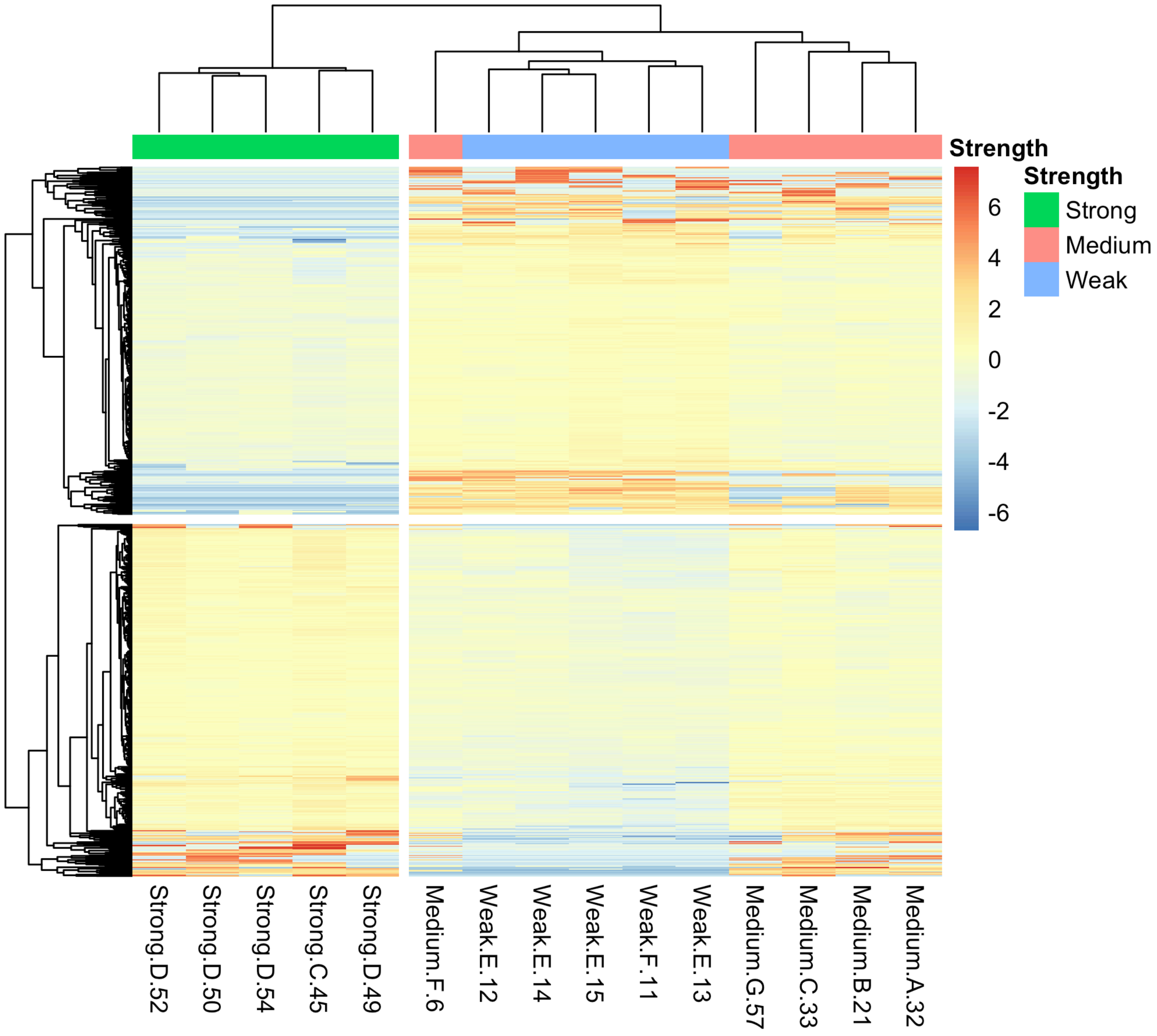

Figure S5 – Heatmap of all 776 (386 upregulated and 390 downregulated of Weak relative to Strong colonies) differentially expressed transcripts by colony. Results were filtered to an adjusted p-value of <0.01. Genes are sorted by standard deviations and plotted values are the difference between LFC and row means. Clustering was performed using complete Euclidean distance.

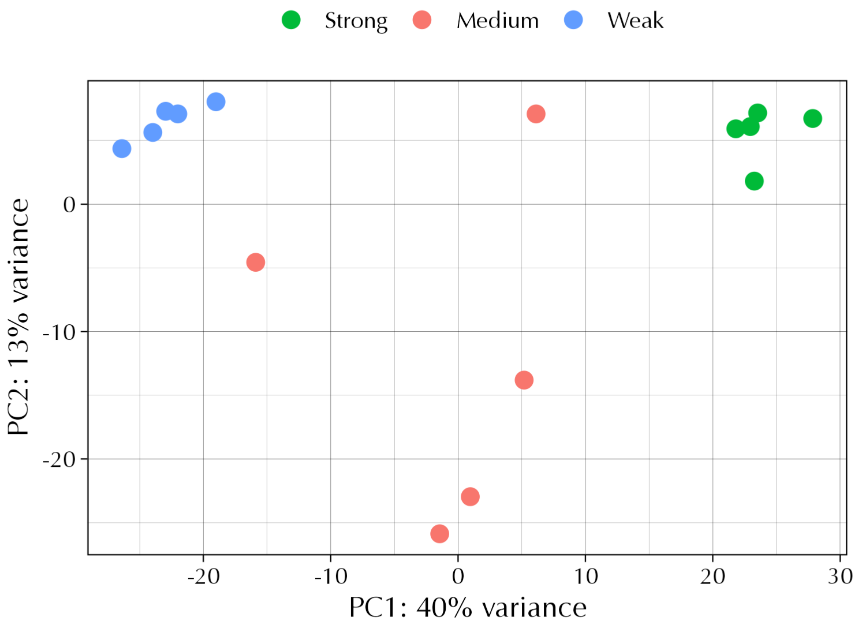

Figure S 6 - Principal component analysis of the top 100 differentially expressed transcripts between colonies of differing population strengths.

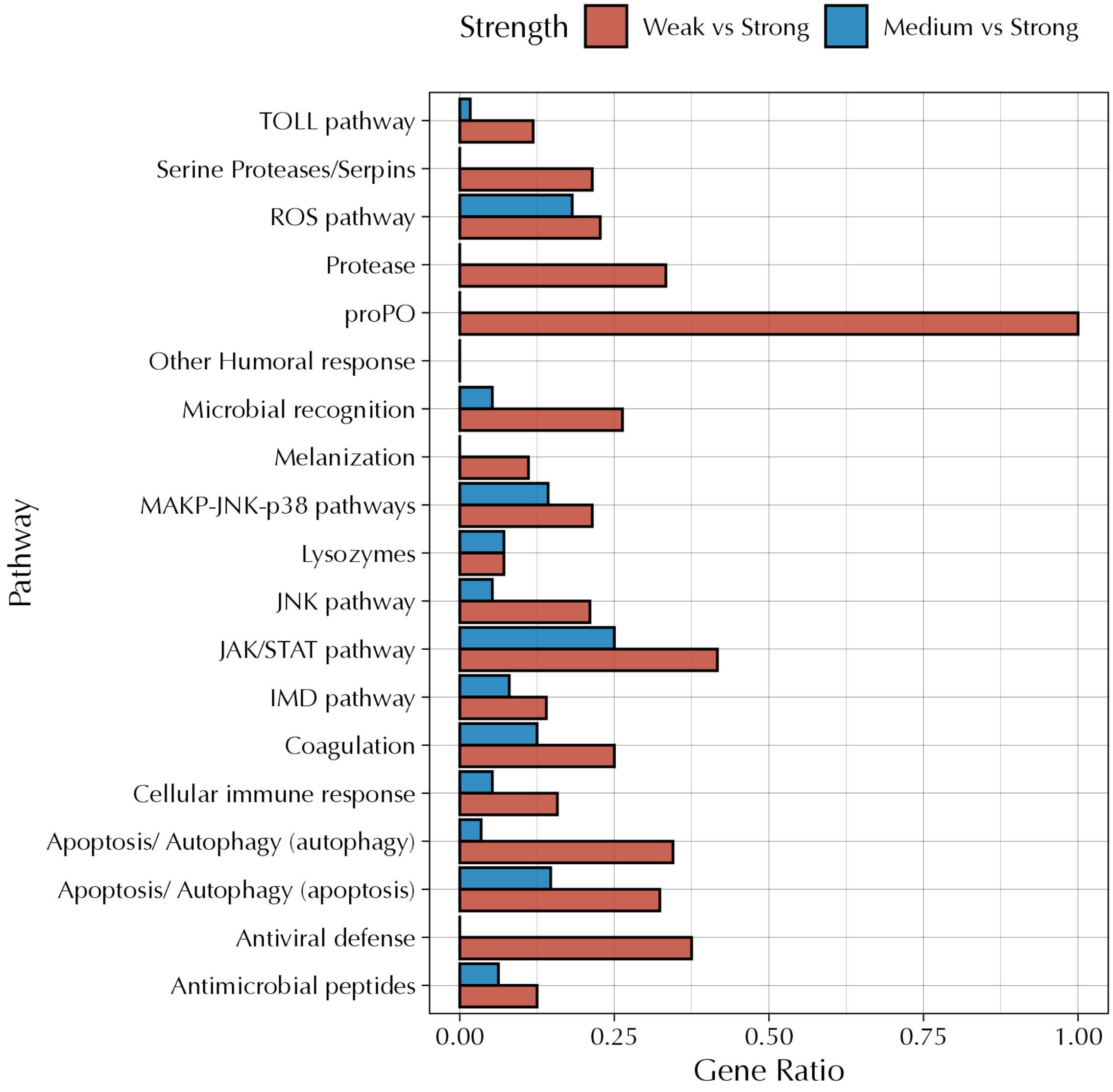

Figure S7 - Ratio of differentially expressed genes by curated immune gene pathway as compared between Weak vs Strong colonies and Medium vs Strong Colonies.

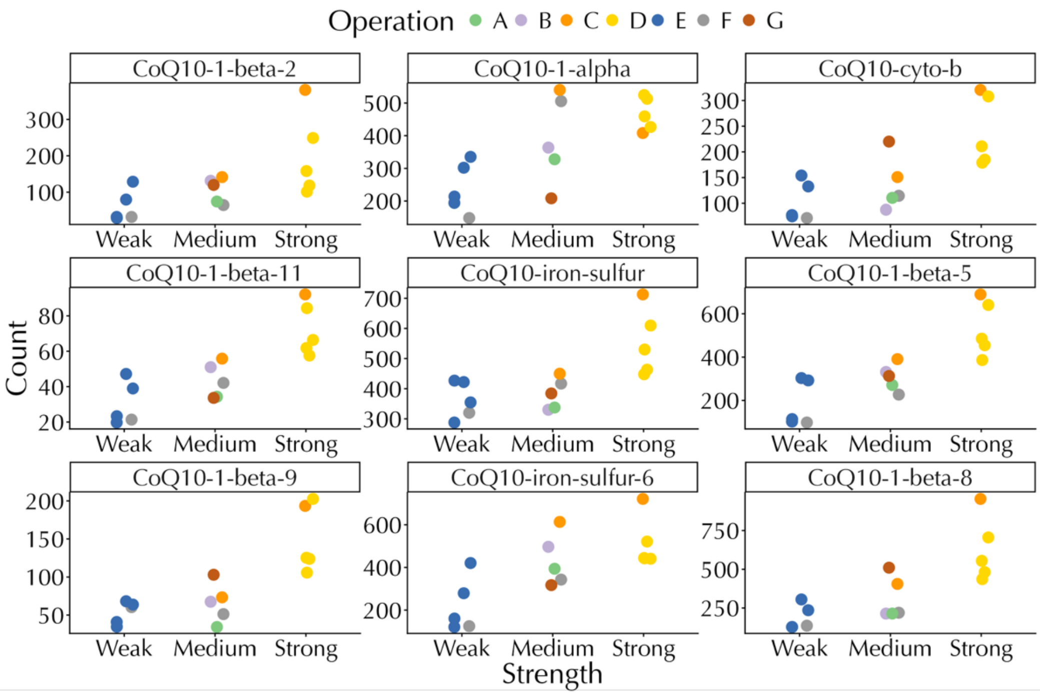

Gene	baseMean	log2FoldChange	lfcSE	stat	pvalue	padj
CoQ10-1-beta-2	122.245	-1.740	0.504	-3.452	0.001	**0.012**
CoQ10-1-alpha	364.160	-0.967	0.276	-3.501	0.000	**0.010**
CoQ10-cyto-b	159.264	-1.234	0.318	-3.874	0.000	**0.003**
CoQ10-1-beta-11	48.146	-1.261	0.286	-4.409	0.000	**0.001**
CoQ10-iron-sulfur	432.436	-0.601	0.165	-3.656	0.000	**0.006**
CoQ10-1-beta-5	339.473	-1.543	0.357	-4.322	0.000	**0.001**
CoQ10-1-beta-9	89.356	-1.487	0.312	-4.761	0.000	**0.000**
CoQ10-iron-sulfur-6	388.845	-1.215	0.356	-3.415	0.001	**0.013**
CoQ10-1-beta-8	374.682	-1.749	0.363	-4.818	0.000	**0.000**

Figure S8 - Coenzyme Q10 (ubiquinone) expression by colony strength.

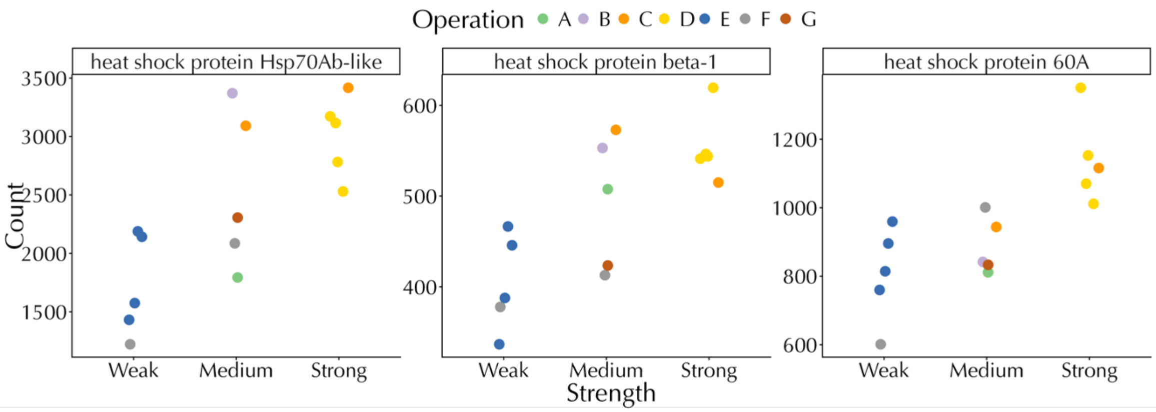

Gene	baseMean	log2FoldChange	lfcSE	stat	pvalue	padj
heat shock protein Hsp70Ab-like	2414.321	-0.811	0.214	-3.783	0	**0.004**
heat shock protein beta-1	482.843	-0.463	0.125	-3.695	0	**0.006**
heat shock protein 60A	943.542	-0.498	0.131	-3.805	0	**0.004**

Figure S9 - Heat shock protein expression by colony strength.

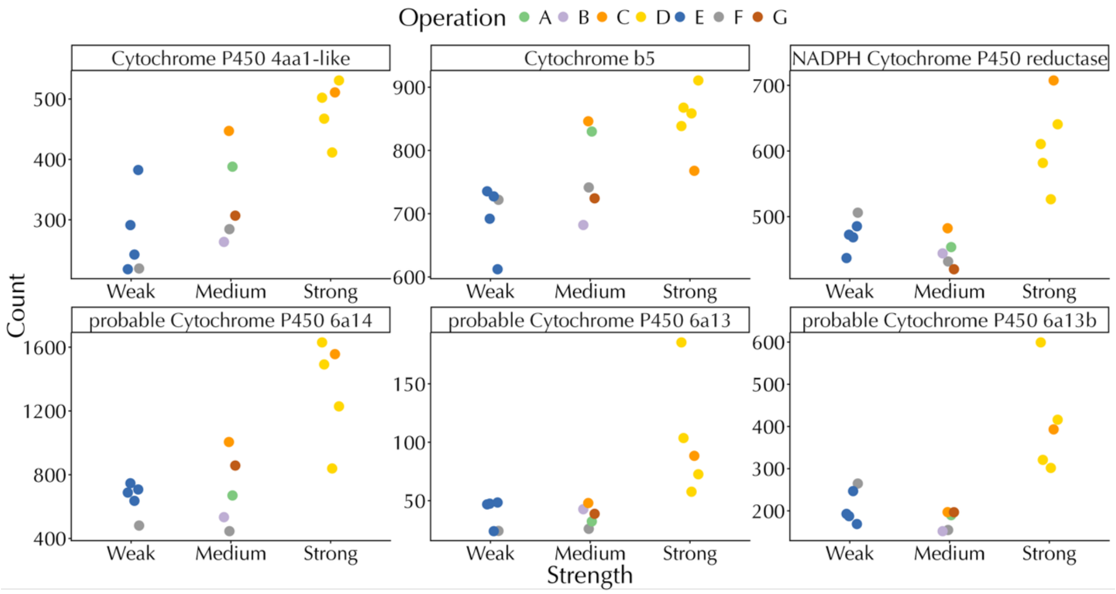

Gene	baseMean	log2FoldChange	lfcSE	stat	pvalue	padj
Cytochrome P450 4aa1-like	363.883	-0.837	0.197	-4.244	0	**0.001**
Cytochrome b5	769.918	-0.288	0.082	-3.514	0	**0.010**
NADPH Cytochrome P450 reductase	510.664	-0.357	0.078	-4.591	0	**0.000**
probable Cytochrome P450 6a14	900.627	-1.049	0.250	-4.205	0	**0.001**
probable Cytochrome P450 6a13	58.561	-1.412	0.353	-4.002	0	**0.002**
probable Cytochrome P450 6a13b	264.912	-0.937	0.205	-4.577	0	**0.000**

Figure S10 - Cytochrome P450 gene family expression by colony strength. Results filtered to an adjusted p-value < 0.01.

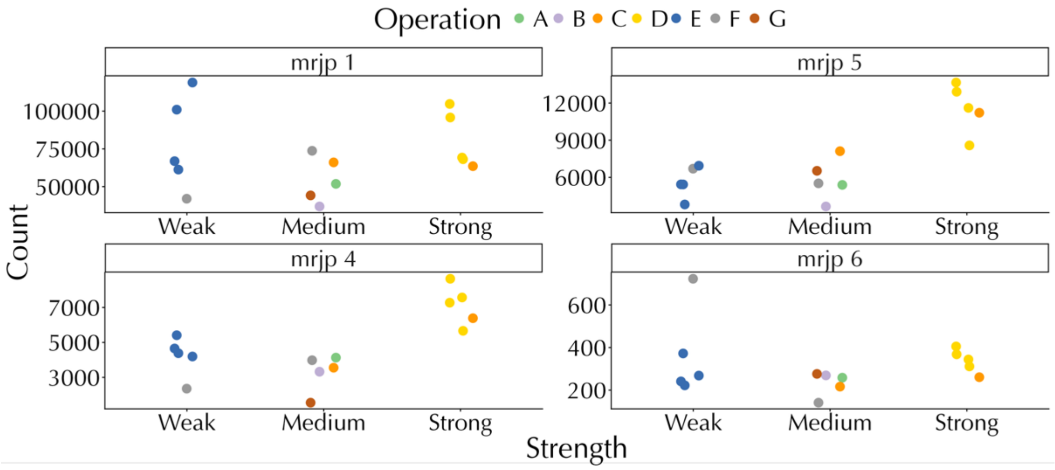

Gene	baseMean	log2FoldChange	lfcSE	stat	pvalue	padj
mrjp 1	70940.620	-0.042	0.297	-0.140	0.888	0.980
mrjp 5	7699.731	-1.033	0.226	-4.572	0.000	**0.000**
mrjp 4	4871.760	-0.759	0.273	-2.775	0.006	0.062
mrjp 6	311.269	0.110	0.328	0.334	0.738	0.948

Figure S11 - Major royal jelly protein expression by colony strength. Gene displayed were selected based on the two highest and two lowest p-values.

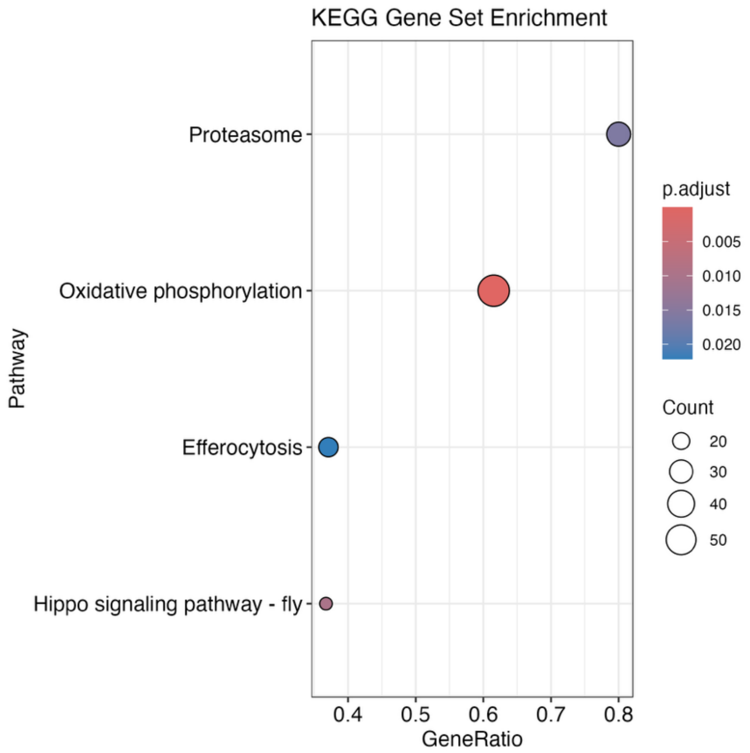

Figure S12 - KEGG Gene Set Enrichment results.

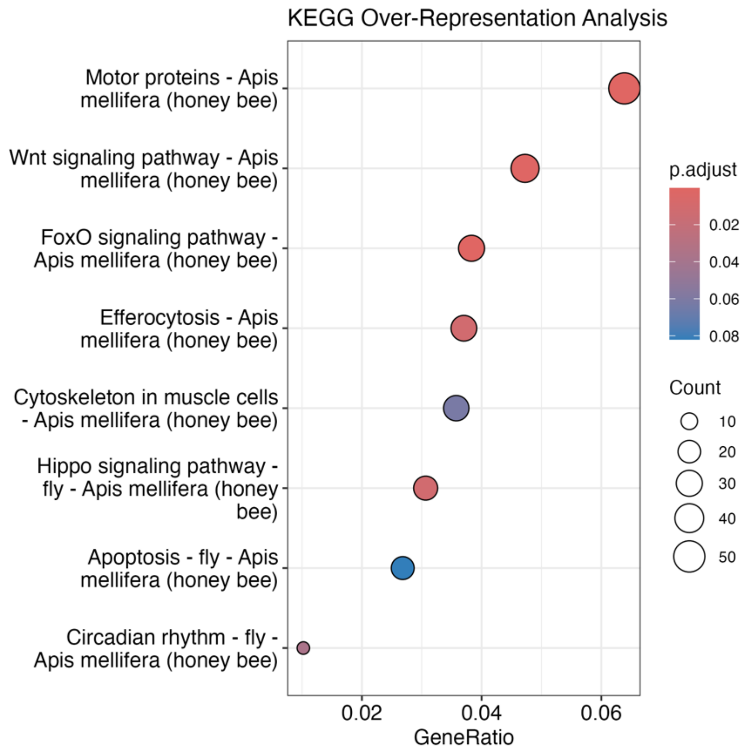

Figure S13 - KEGG Over-Representation results.

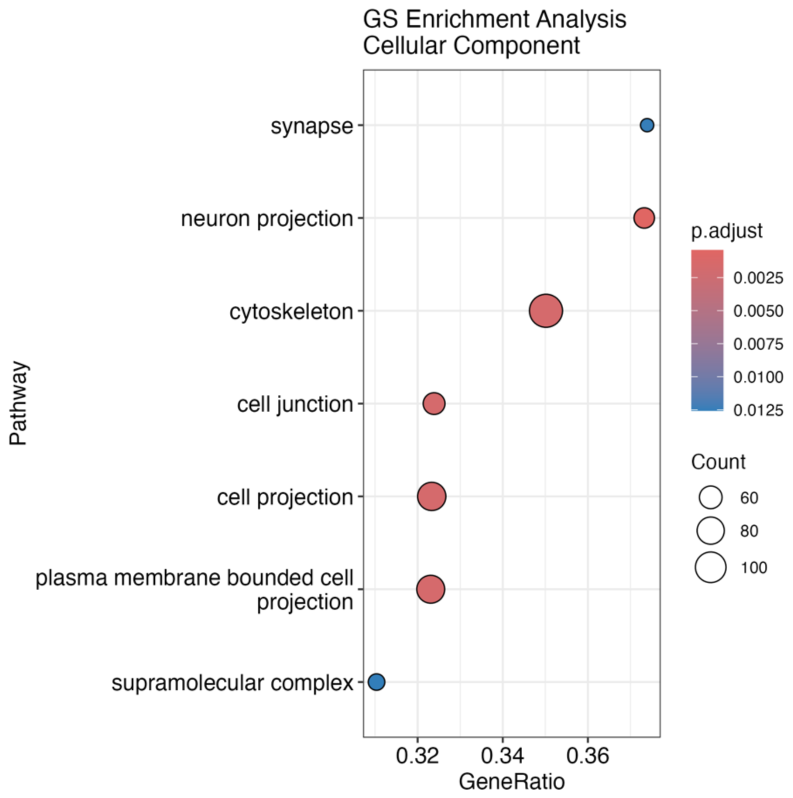

Figure S14 – GO Gene Set Enrichment Analysis for the cellular component.

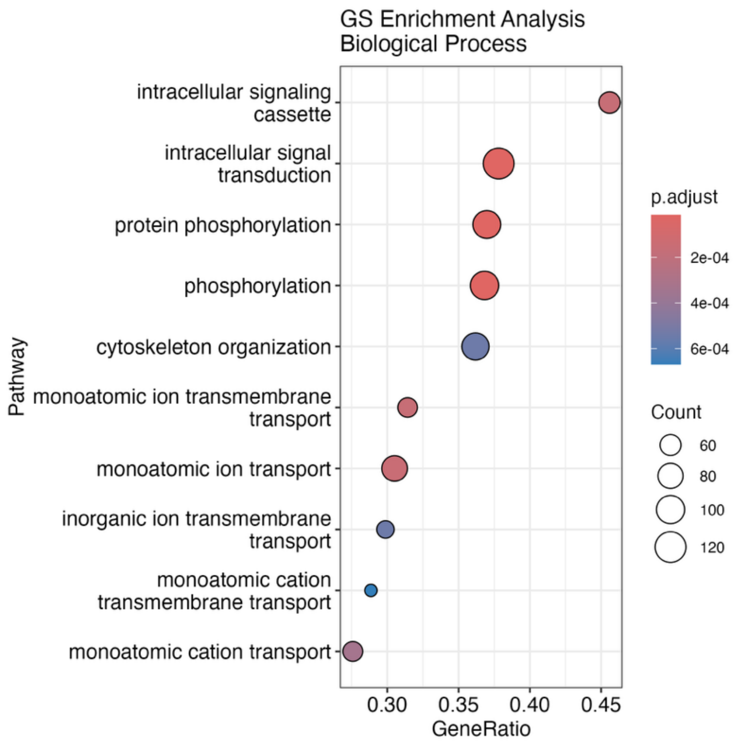

Figure S15 – GO Gene Set Enrichment Analysis for biological component.

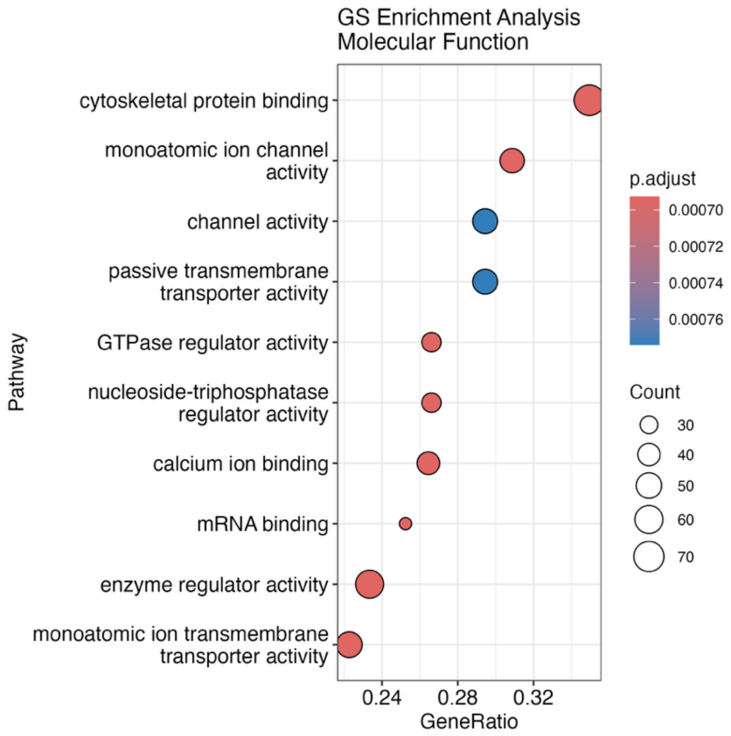

Figure S16 – GO Gene Set Enrichment Analysis for molecular function.

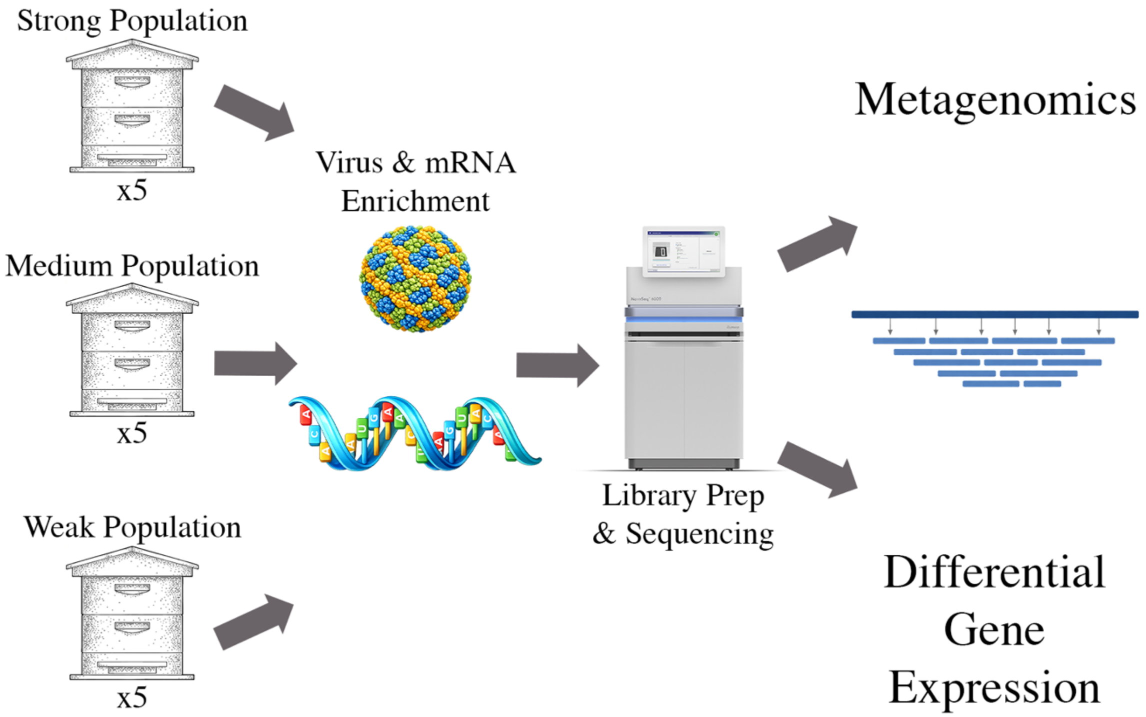

Figure S17 - General experimental design. Colonies were sampled according to their population strengths (n=15) and for each: pooled colony samples were enriched for viruses and mRNA, followed by library preparation and Illumina short read sequencing. Ultimate analyses included viral

metagenomics and differential gene expression. Images depicting virus, mRNA, and read alignment were generated using ChatGPT v5.2. Remaining images are open source.
